# Piezoelectric Bimorph Cantilever for Vibration-Producing-Hydrogen

**DOI:** 10.3390/s130100367

**Published:** 2012-12-27

**Authors:** Jun Zhang, Zheng Wu, Yanmin Jia, Junwu Kan, Guangming Cheng

**Affiliations:** 1 Department of Physics, Zhejiang Normal University, Jinhua 321004, China; E-Mails: maszj8835@126.com (J.Z.); wuzheng@zjnu.cn (Z.W.); 2 College of Geography and Environmental Sciences, Zhejiang Normal University, Jinhua 321004, China; 3 College of Engineering, Zhejiang Normal University, Jinhua 321004, China; E-Mails: kjw@zjnu.cn (J.K.); cgm@zjnu.cn (G.C.)

**Keywords:** piezoelectric, energy harvesting, hydrogen production, piezoelectrochemical

## Abstract

A device composed of a piezoelectric bimorph cantilever and a water electrolysis device was fabricated to realize piezoelectrochemical hydrogen production. The obvious output of the hydrogen and oxygen through application of a mechanical vibration of ∼0.07 N and ∼46.2 Hz was observed. This method provides a cost-effective, recyclable, environment-friendly and simple way to directly split water for hydrogen fuels by scavenging mechanical waste energy forms such as noise or traffic vibration in the environment.

## 1. Introduction

The environmental problems of using fossil fuels have already begun to surface. The excessive use of fossil fuels is one of the primary causes of global warming and acid rain, which have affected the Earth's ecosystem climate and weather conditions [1]. Considering the energy security and global environment, a non-polluting and renewable energy source is urgently needed. Hydrogen, as a clean energy source, presents itself as a potential alternative to fossil fuels with a high calorific value (∼3,042 cals/m^3^) [2] and a high efficiency (∼70%). At present, the main method for hydrogen production is direct water electrolysis, which has the disadvantage of high electricity consumption [3–5]. The electrolytic process is rarely used in industrial hydrogen production since hydrogen can currently be produced more affordably from fossil fuels. The vast majority of hydrogen is produced from hydrocarbons and as a result contains trace amounts of carbon monoxide among other impurities. The carbon monoxide impurity can be detrimental to various systems, including many fuel cells. Extremely high purity hydrogen can be obtained by electrolysis of water, which is environmentally-friendly. Hydrogen intentionally produced from electrolysis is for specific point-of-use applications such as the case with oxyhydrogen torches or when extremely high hydrogen purity or oxygen purity is desired.

Many methods for the front end of hydrogen electrolysis are currently used for electrical production, such as dams, wind-energy and solar energy sources. However, the power from dams, wind-energy and solar energy is limited by location, wind power or light intensity, respectively. In recent years, some novel, high efficient and environment-friendly methods for water-splitting-hydrogen production have been reported [6–11]. The possibility of biological hydrogen production technology with an energy conversion efficiency of ∼28% was raised by Kamen and Gest in 1949 [10,11]. However, its rigorous requirements of light, pH value, temperature and oxygen concentration, made it difficult to see wide application [12–15]. In 1972, Fujishima and Honda reported a simple way to split water into hydrogen by photocatalysis, with an energy conversion efficiency of ∼10%, by using TiO_2_[4]. However, the low energy conversion efficiency limits its practical applications [5–9].

It's well known that the piezoelectric materials can transform mechanical energy into electric energy, which has much higher energy conversion efficiency (up to ∼90%) than other energy conversion (thermoelectric, photovoltaic, magnetoelectric) materials [16]. Mechanical vibration energy is one of the most popular sources of energy in our living environment and is available almost anywhere and anytime [17]. Hydrogen production through water splitting holds particular interest since it utilizes water, an inexpensive renewable resource. Therefore hydrogen production through a vibrating piezoelectric material provides an alternative way to obtain hydrogen energy and has potential for practical application in the future. In 2011, Xu *et al.* reported a way to directly split water for hydrogen production with a ultrahigh energy conversion efficiency of ∼18% by vibrating piezoelectric micro-fibers using a high-frequency ultrasonic wave [18]. In practice, the ambient vibrations in Nature generally occur at low frequencies. A low-frequency piezoelectric hydrogen production device should be designed to better meet the requirements of practical application.

In this article, we fabricated a water splitting device composed of a piezoelectric bimorph cantilever and a water electrolysis setup to realize directly piezoelectrochemical hydrogen production. To obtain the low work frequencies, a piezoelectric bimorph cantilever structure was adopted in our design [19–21].

## 2. Experimental Section

**Figure 1.**
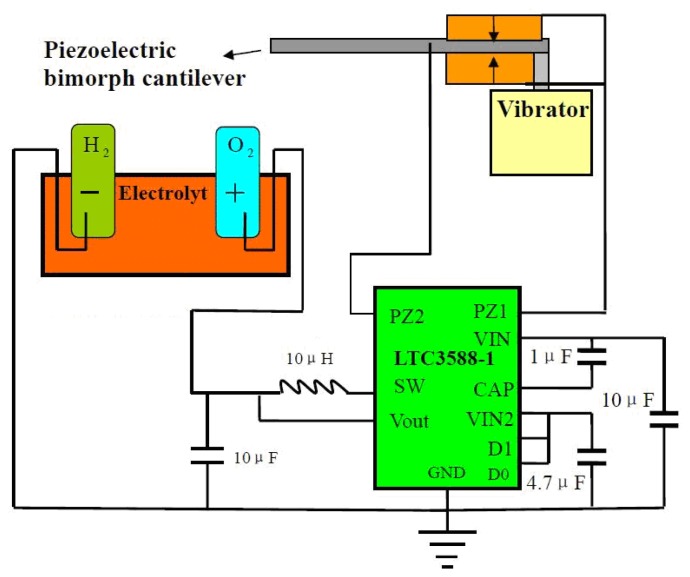
The structure of the hydrogen generation device. Here the volumes of the gas extractor and the electrolysis pool are 0.8 mL and 25 mL, respectively.

Figure 1 illustrates the structure of the hydrogen production device. The working principle can be described as follows: when a cyclic force is applied to the cantilever, the electric potential will occur between the top and the bottom electrode surface of the PZT-5 ceramics due to the piezoelectric effect. The electrical signal produced by the mechanical vibration is rectified and connected with an electrolyte solution to split the water into hydrogen and oxygen.

A PZT-5 ceramic wafer of 30 × 15 × 0.3 mm^3^ size was employed as the piezoelectric layer. The two opposite polarized PZT-5 ceramics and a steel shim with a dimension of 180 × 20 × 0.3 mm^3^ were assembled and bonded together using epoxy conductive Ag glue (Electrolube SCP-03B). The volumes of the gas extractor and the electrolysis pool are about 0.8 mL and 25 mL, respectively. A LTC-3588 chip combined with several capacitors was adopted as the rectifier circuit to transform the output AC signal into a DC one. A pH indicator paper was employed to detect the concentration of the hydrogen ion near the cathode and anode. A vibrator (Model HEV-50, Nanjing Foneng Technology Co., Ltd.) was used to generate a cyclic force of ∼0.07 N. The output voltage from the piezoelectric bimorph cantilever was monitored through an oscilloscope (Tehtronix TDS 2002). A micro gas-collected system was employed to measure the volume of the hydrogen and oxygen produced. It's well known that pure water is a weak electrolyte since it has a low autoionization, *K*_w_ = 1.0 × 10^−14^ at room temperature, and conducts current poorly (0.055 μS·cm^−1^). Unless a very large potential is applied to cause an increase in the autoionization of water, the electrolysis of pure water occurs very slowly or not at all. If a water-soluble electrolyte is added, the conductivity of the water rises considerably. In order to enhance the conductive ionic concentration, here we adopted NaOH and NaHSO_4_ solutions as electrolytes.

## 3. Results and Discussion

**Figure 2.**
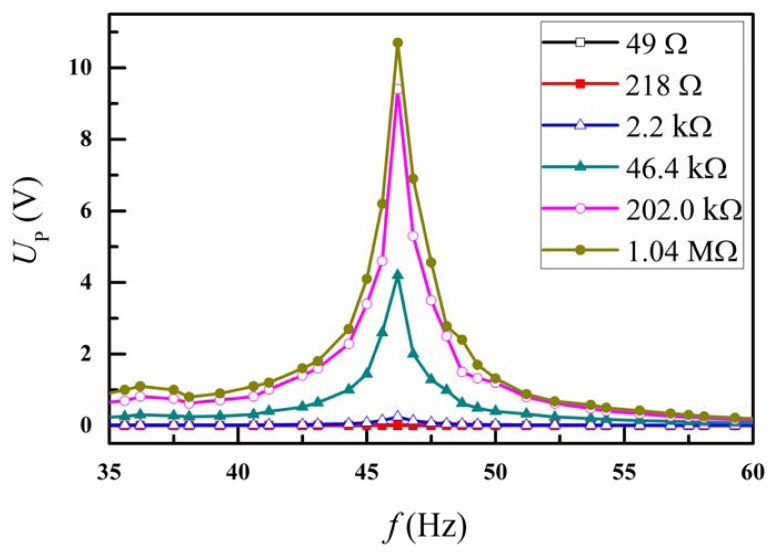
The piezoelectric voltage output of the piezoelectric bimorph cantilever at different electrical resistance load.

Figure 2 showed the piezoelectric voltage output of the piezoelectric bimorph cantilever under different electrical resistance loads. At a resonance frequency of ∼46.2 Hz, the output peak voltage is up to ∼12 V, which is much higher than the standard reduction-oxidation potential of water (∼1.23 V) [18]. Then the electric potential induced by mechanical vibration could easily split the water into hydrogen and oxygen.

**Figure 3.**
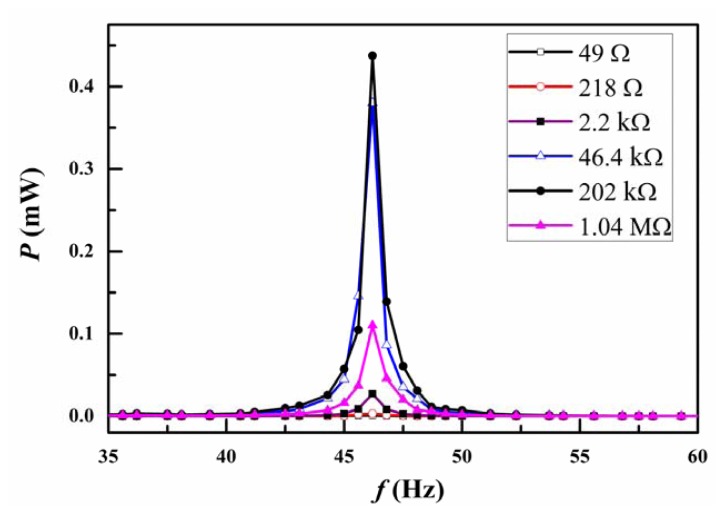
The piezoelectric power output of the piezoelectric bimorph cantilever at different electrical resistance load.

Figure 3 showed the piezoelectric power output of the piezoelectric bimorph cantilever under different electrical resistance loads. Under the excitation of a cyclic force of ∼0.07 N, the maximum power output of the piezoelectric bimorph cantilever is up to ∼0.44 mW at the resonance frequency of ∼46.2 Hz. The matching load resistance for the maximum power output is ∼202 kΩ. In Nature, ambient vibrations generally occur at low frequencies. On basis of the piezoelectric bimorph cantilever structure, we can design a low-frequency vibration-producing-hydrogen device. The maximum piezoelectric voltage output and maximum power output from the piezoelectric bimorph cantilever can be expressed as following [22]:

(1) $$U_{p}={\frac{\omega R_{L}Ah_{13}}{l}p_{0}/\left[{\left(\beta_{11}^{S}\right)}^{2}+{\left(\frac{\omega R_{L}A}{b}\right)}^{2}\right]}^{1/2}$$

(2) $$P={\frac{R_{L}A^{2}h_{13}^{2}p_{0}^{2}}{2l^{2}}/\left[\frac{{\left(\beta_{11}^{S}\right)}^{2}}{\omega^{2}}+\frac{R_{L}^{2}A^{2}}{b^{2}}\right]}^{1/2}$$

where *p_0_*, *b*, *A* and *l* are the vibration vertical displacement, thickness, area, length of the PZT-5 ceramic wafer, respectively. *R_L_* is the load resistance. $h_{13}^{2}$, *ω* and $\beta_{11}^{S}$ are the piezoelectric stiffness coefficient, phase change rate and dielectric isolation rate of the PZT-5 ceramic, respectively.

From Equations (1) and (2), the piezoelectric output voltage under a mechanical vibration is proportional to the piezoelectric coefficient $h_{13}^{2}$ and vibration vertical displacement *p*_0_ of the piezoelectric wafer, while the output power is proportional to $h_{13}^{2}$ and $p_{0}^{2}$. Hence, increasing the piezoelectric coefficient and the vibration vertical displacement of the piezoelectric material can effectively enhance the vibration-producing- hydrogen efficiency.

The conversion efficiency (*η*_1_) from mechanical energy to electric energy for the piezoelectric PZT-5 ceramic plate is about ∼ 36% [22] ( $\eta_{1}=k_{31}^{2}$, where *k*_31_ is the electromechanical coupling factor. For PZT-5 ceramic, *k*_31_ is equal to ∼0.6). The room-temperature energy efficiency of water electrolysis (*η*_2_) is reported as ∼80% [23,24]. Accordingly, on the basis of the principle of product effect [25], the conversion efficiency (*η* = *η*_1_· *η*_2_) from mechanical energy to chemical energy in our vibration-hydrogen producing system can be estimated as ∼28.8%, which is higher than that of photoelectrochemical hydrogen production (the conversion efficiency is ∼6.5%) [26–28].

**Figure 4.**
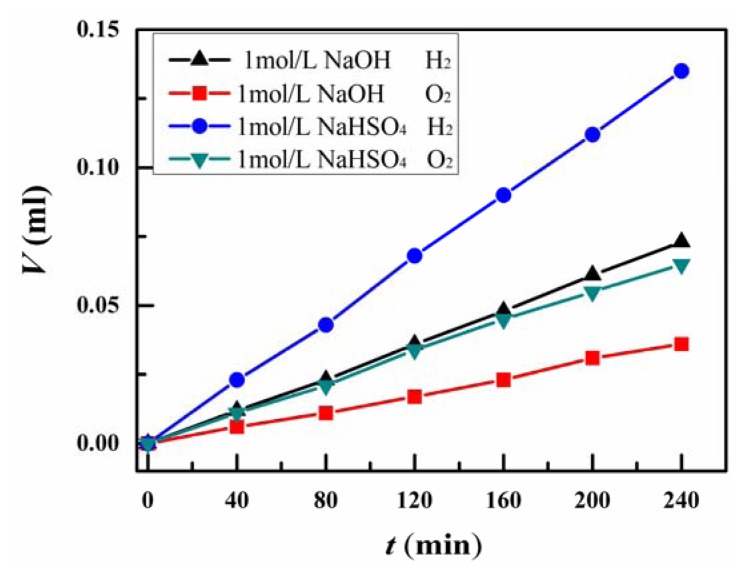
The output volume of the hydrogen and oxygen by the piezoelectric vibration electrolysis of 1 mol/L NaOH and NaHSO_4_ solution.

Figure 4 showed the output volume of the hydrogen and oxygen produced by the low-frequency mechanical vibration as a function of the vibration duration time for 1 mol/L NaOH and 1 mol/L NaHSO_4_ electrolyte solution, respectively. The output volume per unit time of the hydrogen from 1 mol/L NaHSO_4_ solution is twice of that from 1 mol/L NaOH solution. It should be noted that the volume ratio of hydrogen gas and oxygen gas produced by mechanical vibration is equal to 2:1. The water produced by burning of the mix gas of hydrogen and oxygen can be recycled for hydrogen energy production. Then the method of hydrogen production through mechanical vibration has the advantages of high-efficiency, low-cost, environment-free and recyclability.

From Figure 4, the rate of vibration-producing hydrogen in our experiment is much weaker (about 10^−8^ mol/min) than that (∼1.21 mmol/h) of catalytic reactions [27]. The energy conversion efficiency and production rate may be enhanced by adopting the PMN-PT crystal [25], which possesses the highest piezoelectric coefficient (>2,000 pC/N) (the piezoelectric coefficient of the PZT plate used in our current experiment is ∼500 pC/N). Increasing the conductive ion concentration of the NaHSO_4_ electrolyte solution is also helpful for hydrogen production. In practical application, with the increase of vibration electrolysis duration time, the conductive ion concentration will increase. To maintain the continuous output of hydrogen production, water should be added.

Based on our experimental results, the direct conversion from vibration energy to chemical energy was realized by using a piezoelectric PZT-5 ceramics bimorph cantilever to generate H_2_ and O_2_ from water. The piezoelectric effect converts mechanical vibration energy to electrical energy and is a physical process. The piezoelectric PZT plate is reusable and stable in the environment [29]. Continuous mechanical vibration will lead to continuous hydrogen output. Vibrations exist in many circumstances such as tides, wind, bridges, motion. Water is everywhere around us. Therefore hydrogen production through mechanically vibrating piezoelectric materials is a hopeful method for the utilization of recyclable and environment-friendly new energy resource in the future. The device may be used to utilize the vibration energy of tides at the seaside to split seawater for continuous hydrogen production. Compared with other ways of hydrogen production, such as the direct water electrolysis (high electricity consumption) or the biological hydrogen production (rigorous light, pH value, temperature and oxygen concentration conditions), the mechanical vibration piezoelectrochemical hydrogen production technology that we have suggested developing, has the advantages of high-efficiency, low-cost, environment-friendly, simple-technique and recyclability [30].

## 4. Conclusions

In summary, a high efficiency, non-polluting and no electronic power consumption hydrogen generation device was fabricated by using a piezoelectric bimorph cantilever. The obvious output of the hydrogen and oxygen was observed under a mechanical vibration of ∼0.07 N and ∼46.2 Hz. The flexible vibration mode in the piezoelectric bimorph cantilever structure makes the resonance frequency very low. This hydrogen production method provided a cost-effective, recyclable, environment-friendly and simple way to directly split water for hydrogen fuels by scavenging mechanical energy wastes such as noise or traffic vibration in the environment. The device, harvesting environmental vibration waste energy for hydrogen production via piezoelectric effect, may have potential implications in solving the challenges of energy shortages and environmental pollution that we are facing today and in the future.
